# Draft Genome Sequence of the Deep-Sea Bacterium *Shewanella benthica* Strain KT99

**DOI:** 10.1128/genomeA.00210-13

**Published:** 2013-05-30

**Authors:** F. M. Lauro, R. A. Chastain, S. Ferriera, J. Johnson, A. A. Yayanos, D. H. Bartlett

**Affiliations:** School of Biotechnology and Biomolecular Sciences, University of New South Wales, Sydney, New South Wales, Australiaa; Marine Biology Research Division, Scripps Institution of Oceanography, University of California, San Diego, La Jolla, California, USAb; J. Craig Venter Institute, Rockville, Maryland, USAc

## Abstract

We report the draft genome sequence of the obligately piezophilic *Shewanella benthica* strain KT99 isolated from the abyssal South Pacific Ocean. Strain KT99 is the first piezophilic isolate from the Tonga-Kermadec trench, and its genome provides many clues on high-pressure adaptation and the evolution of deep-sea piezophilic bacteria.

## GENOME ANNOUNCEMENT

Members of the genus *Shewanella* are Gram-negative gammaproteobacteria that are common in aquatic environments and are well known for their multifaceted respiration systems. Despite their distribution, only 2 species are found in ocean waters deeper than 2,000 m: *Shewanella benthica* and *Shewanella violacea* (1). These isolates are usually piezophilic and psychrophilic (2, 3).

On 17 October 2001, an insulated trap baited with scraps of mahi-mahi was deployed as a free vehicle into the Kermadec Trench at 32° 01.07’S, 177° 20.99'W over a depth of 9,856 m for 14 h. The temperature at that depth was ~1.8°C, and recovered trapped amphipods (dead from decompression) were found at <6°C and were immediately moved to a cold room. Several amphipods were heat sealed in plastic bags and compressed to 99.7 MPa (4). One of these bags with decayed amphipods was used, after completion of the cruise, to grow bacterial colonies using methods previously described (4) at 99.7 MPa and ~2°C. One of the colonies was dubbed strain KT99.

Freshly inoculated cultures (1:1,000 dilution) of *S. benthica* KT99 reach the stationary phase in ~160 h at 10°C and 90 MPa. No growth is observed at the same temperature at 40 MPa or 140 MPa.

Genomic DNA was purified from 1.5 liters of culture grown at 90 MPa and 10°C. The cells were harvested, washed once in phosphate-buffered saline (PBS), and resuspended in 2.4 ml of nuclei lysis buffer (Promega). The extraction was completed according to the protocol of the Wizard Genomic DNA kit (Promega). The precipitated DNA pellet was further purified by resuspension in 1.2 ml of of Tris-EDTA (TE) buffer, extraction once with 1.2 ml of phenol-chloroform (pH 8.0) and once with 1.2 ml of chloroform, and precipitation by centrifugation (16,000 × *g*, 10 min, 4°C) with 120 µl of sodium acetate (3 M [pH 4.8]) and 4 ml of ethanol (100%). A small aliquot was used for quantification and quality control.

The draft genome sequence was determined by shotgun sequencing as follows: two genomic libraries with insert sizes of 4 kb (plasmid) and 40 kb (fosmid) were constructed. The prepared plasmid and fosmid clones were sequenced from both ends to provide paired-end reads on ABI 3730xl DNA sequencers (Applied Biosystems). Successful reads were used as input values for the Celera Assembler. The whole-genome shotgun (WGS) sequence produced by the assembler was then annotated using the PGAAP at NCBI.

The genome of *S. benthica* KT99 has 4.35 million bp encoding 4,235 predicted open reading frames (ORFs), with a G+C content of 46%. There are multiple predicted copies of the rRNA operon that carry elongated stem-loops found only in piezophilic bacteria (5).

The genome contains complete pathways for a heterotrophic lifestyle, such as the complete glycolytic and tricarboxylic acid (TCA) pathways. The COG composition compared to that of the sister species *Shewanella frigidimarina* NCIMB400 (accession no. NC_008345) was found to be statistically enriched in the genes for DNA replication, recombination, and repair (COG category L) and, in particular, for 4 different classes of transposases (COG2826, COG3436, COG3676, COG5433). Deep-sea metagenomes are overrepresented in mobile elements (6, 7) that might have functional roles in the hadal and abyssal ocean (7).

The genome also encoded a type A fatty acid synthase-polyketide synthase (FAS-PKS) system (8) for the synthesis of polyunsaturated fatty acids, such as eicosapentaenoic acid (EPA) (20:5, n-3) and docosahexaenoic acid (DHA) (22:6, n-3). *S. benthica* KT99 has been shown to produce EPA and traces of DHA (9), and the resulting increase in membrane unsaturation might play a role in low-temperature and high-pressure environments (10). There was no evidence for the presence of light-activated photolyases, a hallmark for autochthony in deep-sea bacteria (9).

### Nucleotide sequence accession number.

The Whole-Genome Shotgun project was deposited in NCBI under the accession no. ABIC00000000.
